# Cost-effectiveness of implementing a digital psychosocial intervention for patients with psychotic spectrum disorders in low- and middle-income countries in Southeast Europe: Economic evaluation alongside a cluster randomised trial

**DOI:** 10.1192/j.eurpsy.2022.2310

**Published:** 2022-08-26

**Authors:** Y. Feng, C. Roukas, M. Russo, S. Repišti, A. Džubur Kulenović, L. Injac Stevović, J. Konjufca, S. Markovska-Simoska, L. Novotni, I. Ristić, E. Smajić-Mešević, F. Uka, M. Zebić, L. Vončina, A. Bobinac, N. Jovanović

**Affiliations:** 1 Wolfson Institute of Population Health, Queen Mary University of London, London, UK; 2 Psychiatric Clinic, Clinical Centre of Montenegro, Podgorica, Montenegro; 3 Clinical Center University of Sarajevo, Sarajevo, Bosnia and Herzegovina; 4 Department of Psychology, University of Pristina, Pristina, Kosovo by United Nations Resolution; 5 Macedonian Academy of Sciences and Arts, Skopje, Republic of North Macedonia; 6 University Clinic of Psychiatry, Skopje, Republic of North Macedonia; 7 Faculty of Medicine, University of Belgrade, Belgrade, Serbia; 8 Faculty of health studies, University of Rijeka, Rijeka, Croatia; 9 Center for Health Economics and Pharmacoeconomics, Faculty of Economics and Business, University of Rijeka, Rijeka, Croatia

**Keywords:** Cluster randomised trial, Cost-effectiveness, DIALOG+, Low- and middle-income countries in Southeast Europe, Psychotic disorders

## Abstract

**Background:**

DIALOG+ is a digital psychosocial intervention aimed at making routine meetings between patients and clinicians therapeutically effective. This study aimed to evaluate the cost-effectiveness of implementing DIALOG+ treatment for patients with psychotic disorders in five low- and middle-income countries in Southeast Europe alongside a cluster randomised trial.

**Methods:**

Resource use and quality of life data were collected alongside the multi-country cluster randomised trial of 468 participants with psychotic disorders. Due to COVID-19 interruptions of the trial’s original 12-month intervention period, adjusted costs and quality-adjusted life years (QALYs) were estimated at the participant level using a mixed-effects model over the first 6 months only. We estimated the incremental cost-effectiveness ratio (ICER) with uncertainty presented using a cost-effectiveness plane and a cost-effectiveness acceptability curve. Seven sensitivity analyses were conducted to check the robustness of the findings.

**Results:**

The average cost of delivering DIALOG+ was €91.11 per participant. DIALOG+ was associated with an incremental health gain of 0.0032 QALYs (95% CI –0.0015, 0.0079), incremental costs of €84.17 (95% CI –8.18, 176.52), and an estimated ICER of €26,347.61. The probability of DIALOG+ being cost-effective against three times the weighted gross domestic product (GDP) per capita for the five participating countries was 18.9%.

**Conclusion:**

Evidence from the cost-effectiveness analyses in this study suggested that DIALOG+ involved relatively low costs. However, it is not likely to be cost-effective in the five participating countries compared with standard care against a willingness-to-pay threshold of three times the weighted GDP per capita per QALY gained.

## Introduction

The international prevalence of psychotic disorders is approximately 0.75% [1], and the life expectancy of people with psychosis is 10–15 years shorter than the general population [2]. These illnesses are usually associated with poor quality of life and multi-morbidity [3]. They also often lead to high societal costs, including direct costs for patients’ healthcare and costs related to productivity losses [4]. In low- and middle-income countries (LMICs) in Southeast Europe, an estimated 45% of patients with psychotic disorders have experienced a treatment gap (i.e., difference between the treatment they require and the treatment they receive) [5–7]. This is the result of shortages in funding and qualified staff, and a high patient load. Reducing the treatment gap in those countries through the implementation of effective and low-cost interventions is an urgent need.

DIALOG+ is an app-based psychosocial therapeutic intervention. Previously, interactions between psychotic patients and clinicians in routine face-to-face clinical meetings were guided solely by clinical judgment rather than evidence-based methods [8, 9]. DIALOG+ was originally developed to make meetings therapeutically effective [8]. To do this, the intervention implements a structured self-assessment for patients during the meetings and provides guidance for clinicians on how to respond to patients’ ratings. Previous studies have shown that using DIALOG+ is effective in improving the quality of life for patients with psychosis in UK community-based settings [8, 10]. Furthermore, the effectiveness of DIALOG+ has been extensively studied in mental health care across multiple countries and in different healthcare settings [11–14].

As DIALOG+ is used in existing routine patient–clinician meetings, it does not require the formation of new services or hiring of new staff, and only requires that the existing service makes a one-off investment in computer tablets. The intervention can then be widely used by clinicians with minimal training, making it a good fit for healthcare systems with scarce resources [15]. Evidence from high-income settings suggests that DIALOG+ is a cost-saving intervention for people with mental disorders [10]. The intervention also has the potential to deliver benefits for psychotic patients in low-resource settings. However, no study has previously evaluated its implementation in LMICs in Southeast Europe. A multi-country cluster randomised trial within the IMPULSE study was conducted to fill this empirical gap. The trial aimed to evaluate the effectiveness and cost-effectiveness of implementing DIALOG+ in five LMICs in Southeast Europe compared to standard care for patients with psychotic disorders [15].

The primary aim of this paper is to report the cost-effectiveness analyses of the DIALOG+ intervention versus standard care carried out in five Southeast European countries alongside the cluster randomised trial within the IMPULSE study.

## Methods

### Trial design

The cluster randomised trial within the IMPULSE study recruited participants from five Southeast European countries: Bosnia and Herzegovina, Kosovo (UN Resolution), Montenegro, North Macedonia, and Serbia. These countries shared similar socioeconomic and political backgrounds before the 1990s, which facilitated the trial setup and mutual learning across sites [15]. Eligible participants were identified through a review of medical records. Participants were eligible if they had: a primary diagnosis of psychosis or related disorder in remission with ICD-10 code F20-29 or F31; a lifetime history of being admitted to hospital at least once; a record of attending outpatient psychiatric services; and the capacity to provide written informed consent. Participants with diagnoses of organic brain disorders and/or severe cognitive deficits were excluded from the trial. Clinicians were randomised to either the intervention group (DIALOG+) or control group (standard care). Details about the trial methodology and implementation of the intervention can be found in the trial protocol [15]. The trial was launched in March 2019 and completed in July 2020.

### DIALOG+ intervention and standard care

#### DIALOG+ intervention

DIALOG+ is a full therapeutic intervention which aims to make existing routine patient–clinician meetings therapeutically effective. The intervention is based on the quality of life research, and embeds the concepts of a patient-centered approach and solution-focused therapy in order to provide an evidence-based structure to routine clinical meetings between patients and clinicians. The intervention consists of two parts: (1) a patient self-rating exercise of satisfaction with their life and treatment, followed by (2) a four-step solution-focused discussion that aims to address the patients’ concerns and agree on further actions.

The trial was designed so that participants in the intervention group would receive six sessions of treatment during their routine outpatient consultations over a 12-month period. In accordance with the DIALOG+ manual [16], each session lasted between 30 and 60 minutes. In the first 3 months, participants received one session per month, followed by one session every 3 months.

Every intervention session started with the patient self-rating their satisfaction with eight life domains (mental health, physical health, job satisfaction, accommodation, leisure activities, partner/family, friendships, personal safety) and three treatment domains (medication, practical help, meetings with clinician) using the DIALOG+ app installed in computer tablets. Next, clinicians were instructed to provide positive feedback to patients for any domain that was scored highly by patients and (from session two onwards) for domains with an improvement in rating from previous sessions. After the self-rating exercise, clinicians and patients identified a maximum of three domains for discussions. These discussions were guided by a four-step approach based on the principles of solution-focused therapy. Finally, the patients and clinicians jointly agreed on actions to improve the patients’ satisfaction with the discussed domain(s). At the beginning of the next session, they reviewed those actions together [17]. Each clinician in the intervention group received face-to-face training by a local research team member before the first DIALOG+ session, followed by top-up training after delivering the third session. Clinicians were also able to access individual supervision provided by the study researchers after each session. A computer tablet with DIALOG+ installed was offered to each clinician prior to the first session.

#### Standard care

Standard care included consultations on medication, psychological support, and discussion with patients on other aspects of care. Participants receiving standard care were offered six sessions of treatment over the 12-month trial period following the same delivery schedule as participants in the intervention group.

### Impact of the COVID-19 pandemic

Although the trial intervention was originally designed to last 12 months, interruption due to the COVID-19 pandemic from March 2020 onward led to significant changes in the intervention, patient assessments, data collection, and retention in the last stage of the trial [14]. Only Serbia completed the six sessions and the last assessment (at month 12) as per protocol before the introduction of local restrictions. The other four countries adapted the DIALOG+ manual, and delivered the last two sessions (fifth and sixth) and the last assessment remotely. Because of these changes, the effect of the complete intervention at 12 months (i.e., six sessions) could not be explored. Therefore, the economic evaluation was based on the first 6 months of trial data (first four sessions), starting from the implementation of the intervention at baseline.

### Study measures

#### Outcome measures

Three instruments were used to assess the quality of life of participants, including the 5L version of the EQ-5D (EQ-5D-5L) [18], Manchester Short Assessment of Quality of Life (MANSA) [19], and the 10-item version of Recovering Quality of Life (ReQoL-10) [20]. Due to the COVID-19 pandemic (see section title “Impact of the COVID-19 pandemic”), only data collected at baseline and 6 months after randomisation were used in the analysis.

The EQ-5D-5L measured the primary economic evaluation outcome. EQ-5D-5L data were converted to index scores by applying the EQ-5D-5L value set. There was no country-specific value set available for any of the five participating countries, so we applied the newly published EQ-5D-5L value set for Poland [21] in Central Europe as the best proxy available. Quality-adjusted life years (QALYs) for participants during the first 6-month period of the trial were calculated using the area-under-the-curve method and EQ-5D-5L index scores [22]. MANSA measured the primary clinical effectiveness outcome in the IMPULSE trial. MANSA scores were calculated as the mean of the instrument’s 12 individual item scores. ReQoL-10 is a new instrument for measuring the quality of life in people with mental health conditions. For ReQoL-10 data, simple sum scores on the instrument’s 10 questions were calculated.

For all three outcome measures, lower score indicates poorer quality of life. EQ-5D-5L index scores have a theoretical range between −0.590 and 1. The range is 1 to 7 for MANSA scores, and 0 to 40 for ReQoL-10 scores.

#### Costs data

The retrospective costs data 6 months prior to baseline and 6 months after randomisation were collected using an adapted version of the Client Service Receipt Inventory (CSRI) [23]. The CSRI recorded participants’ use of inpatient hospital services, community care service, primary care service, and medication. We collected unit costs for each item from the local teams in the five participating countries. Data on participants’ socio-demographics, employment status, monthly income, number of days off from work due to mental and/or physical health issues, amount of state benefits claimed, and criminal records were also collected using the CSRI.

We developed a health economics inventory form to collect cost data for providing DIALOG+ and standard care treatments. Items included time spent by clinicians on the DIALOG+ training, time spent by clinicians and supporting staff on treatments, quantity of equipment and key materials used for providing treatments. We also collected the unit cost for each item using the inventory form.

We converted all unit costs from local currencies to euros at the year 2019 level with Purchasing Power Parity (EU28 = 1 as the reference base) adjusted [24]. Costs for each item were then calculated as a product of the quantity used and its corresponding unit cost. Finally, we summed all costs together and presented the cost data at participant and assessment time-point levels. There was no discount applied to adjust costs and outcomes data as the time horizon of the study was 6 months [25].

Outcome and cost measures used in the economic evaluation are validated scales, including EQ-5D-5L [18], MANSA [19], ReQoL-10 [20] and CSRI [23]. They were translated into the local languages by study researchers from central and local research teams before being administered to participants.

### Economic evaluation

We compared participant-level costs and outcomes data between the two trial groups at each assessment time point (i.e., baseline and 6 months after randomisation). Independent *t*-tests were used for all comparisons. The 95% confidence intervals (CIs) were constructed using a bootstrap method with 1,000 replications. We also applied a three-level mixed-effects model to recognise the clustered nature of our data where participants nested within clinicians that nested within countries. The model controlled for baseline variables (i.e., costs or outcomes) and covariates (i.e., age of participants, ICD-10 code, and profession of clinicians).

We conducted the within-trial analyses from a healthcare perspective under the principle of intention-to-treat. Time horizon for the economic evaluation was 6 months, starting from the implementation of the intervention at baseline. This was consistent with the time horizon for the effectiveness evaluation of DIALOG+ in the IMPULSE trial [14].

Cost–utility analysis was used to conduct the base case economic evaluation. Costs included intervention costs, health service costs, and medication costs. The primary economic outcome measure used QALYs calculated from the EQ-5D-5L index scores. We estimated the incremental costs (and incremental QALYs) as the difference between the intervention and control groups over the first 6 months of the trial period, controlling for baseline values, participants’ ages, ICD-10 code, and profession of clinicians. A three-level mixed-effects model was applied. The pattern of missing values with three variables (i.e., costs at baseline, costs, and QALYs over the 6-month period) was assumed as missing at random. Multiple imputation with chained equations was applied to generate 70 imputed data sets (the largest fraction of missing information was 0.5258). The point estimate of the incremental cost-effectiveness ratio (ICER) was calculated by dividing the estimated incremental costs by the estimated incremental QALYs. To explore the uncertainty around the point estimate, we used the non-parametric bootstrap approach with 1,000 replications to estimate the 95% CI around the ICER [26]. The result was presented using a cost-effectiveness plane. We also constructed a cost-effectiveness acceptability curve to show the probability that DIALOG+ was cost-effective compared with standard care for a range of willingness-to-pay values for an additional QALY gained.

There is no evidence-based cost-effectiveness threshold to apply in multi-country trials for LMICs [27]. The World Health Organization has recommended using one to three times the gross domestic product (GDP) per capita of an LMIC as the cost-effectiveness threshold for the country [28, 29]. An intervention with an estimated ICER of less than three times the national annual GDP per capita is considered cost-effective. In our base case evaluation, we compared our point estimate of the ICER against one to three times the weighted GPD per capita. The weights are proportions of participants from each country out of the total trial sample size.

To check the robustness of the findings from the base case evaluation, we conducted seven sensitivity analyses. First, we ran the base case analysis with complete cases only (i.e., without missing values). Second, the seemingly unrelated regression model without robust standard error was applied to compare the impact of the model choice [30]. Third, we estimated two ICERs using the minimum (and maximum) unit costs, respectively, for all medications from each country when unit costs for some medications were reported in a range. Fourth, we undertook analyses using a broader analytical perspective, including costs due to productivity lost as a result of mental or physical health problems. In the fifth and sixth sensitivity analyses, we replaced the outcome measure EQ-5D-5L index scores with MANSA scores and ReQoL-10 sum scores, respectively. Finally, we estimated country-specific ICERs by applying the method developed by Willke and colleagues [31].

Statistical significance was determined at the 5% level (*p* < 0.05). All analyses were performed with the software package STATA/MP 17 [32].

## Results

### Characteristics of the sample

We present the characteristics of all participants at baseline in Table 1. In total, 468 eligible participants were recruited, with 236 receiving the DIALOG+ treatment and 232 receiving standard care. There were 424 participants at 6 months after randomisation. The trial recruited 81 clinicians from 11 clinics across five countries. The average age of participants in the trial was 42.59 years old (standard deviation [SD] = 11.30). More than half of the participants were male (54.3%), single (54.3%), unemployed (59.7%), not receiving any state benefits (56.8%), and reported the highest level of education as high school (60.5%). Montenegro contributed the largest trial sample (*n* = 122, 26.1%), followed by Kosovo (UN Resolution; *n* = 103, 22%), North Macedonia (*n* = 82, 17.5%), Bosnia and Herzegovina (*n* = 81, 17.3%), and Serbia (*n* = 80, 17.1%).

**Table 1.** Baseline characteristics of participants by trial group for five participating countries.

	DIALOG+ intervention (*N* = 236)	Standard care (*N* = 232)	Overall sample (*N* = 468)
Age in years (mean, SD)	44.34 (11.09)	40.81 (11.26)	42.59 (11.30)
Sex (% female)	103 (43.64%)	111 (47.84%)	214 (45.73%)
Countries (*N*, %)			
Bosnia and Herzegovina	40 (16.95%)	41 (17.67%)	81 (17.31%)
Kosovo (UN Resolution)	52 (22.03%)	51 (21.98%)	103 (22.01%)
Montenegro	62 (26.27%)	60 (25.86%)	122 (26.07%)
North Macedonia	41 (17.37%)	41 (17.67%)	82 (17.52%)
Serbia	41 (17.37%)	39 (16.81%)	80 (17.09%)
Marital status (*N*, %)			
Single	121 (51.27%)	133 (57.33%)	254 (54.27%)
Married/co-living/any partnership	66 (27.97%)	59 (25.43%)	125 (26.71%)
Separated/divorced	38 (16.10%)	37 (15.95%)	75 (16.03%)
Widow/widower	11 (4.66%)	3 (1.29%)	14 (2.99%)
Educational level (*N*, %)			
Less than elementary school	2 (0.85%)	7 (3.02%)	9 (1.92%)
Elementary-school graduate	49 (20.76%)	30 (12.93%)	79 (16.88%)
High-school graduate	139 (58.90%)	144 (62.07%)	283 (60.47%)
University/college graduate	40 (16.95%)	45 (19.40%)	85 (18.16%)
Postgraduate/professional qualification	4 (1.69%)	4 (1.72%)	8 (1.71%)
Other qualification	2 (0.85%)	2 (0.86%)	4 (0.85%)
Employment status (*N*, %)^a^			
Paid employment	29 (12.29%)	39 (16.81%)	68 (14.56%)
Sheltered employment	1 (0.42%)	1 (0.43%)	2 (0.43%)
Training/education	7 (2.97%)	13 (5.60%)	20 (4.28%)
Unemployed	140 (59.32%)	139 (59.91%)	279 (59.74%)
Retired	54 (22.88%)	39 (16.81%)	93 (19.91%)
Other	4 (1.69%)	1 (0.43%)	5 (1.07%)
State benefits (*N*, %)^b^			
No	128 (54.24%)	138 (59.48%)	266 (56.84%)
Yes	106 (44.92%)	90 (38.79%)	196 (41.88%)

a There is one observation missing in the DIALOG+ group with *N* = 235.

b There are two observations missing in the DIALOG+ group with *N* = 234 and four observations missing in the standard care group with *N* = 228.

### Costs for DIALOG+ and standard care interventions

The average cost of delivering DIALOG+ for each participant was €91.11 during the 6-month trial period. The majority of this cost was for clinicians’ time, with €50.92 spent on delivering DIALOG+ and €14.69 on training. The cost also included key resource use (€17.66; computer tablets, fee for translating DIALOG+ manual to local language, room booking for DIALOG+ training), and other equipment use (€6.59; cell phones, recording devices, stationery). Costs from other staff that supported the delivery of DIALOG+ were minor at €1.24 per participant. The average total cost for delivering standard care sessions during the 6-month trial period was €20.87 per participant.

### Resource use and costs

Table 2 presents the quantity of resource use at the participant level over the 6-month trial period, while Supplementary Appendix 1 reports the unit costs for each resource use item. Table 3 shows the average cost per participant for resource use over the 6-month trial period. The single most costly resource was medication. On average, the medication cost for participants in the intervention group was €237.23 per participant, while the average medication cost in the control was €243.35. The total cost in the intervention group was €565.95 per participant and €497.78 per participant in the control. The difference in total cost between the groups was €68.17 (95% CI –54.26, 168.60), but this was not statistically significant as suggested by an independent *t-*test. While controlling for the differences in total costs and the list of other covariates at baseline, the mixed-effects models produced qualitatively similar results. The difference in total cost was estimated as €98.42 (95% CI –29.49, 208.30), although this was not statistically significant.

**Table 2.** Mean resource use in quantities over the first 6 months of the trial by group.

	DIALOG+ intervention (*N* = 236)		Standard care (*N* = 232)	
	*N* ^a^	Mean (SD)	*N* ^a^	Mean (SD)
		[min, max]		[min, max]
Inpatient service				
Voluntary admission to psychiatric hospital (days)	206	2.00 (14.15)	218	1.20 (7.03)
		[0, 180]		[0, 60]
Involuntary admission to psychiatric hospital (days)	206	0.54 (4.41)	218	0.06 (0.62)
		[0, 54]		[0, 7]
Admission to hospital for physical health (days)	206	0.19 (1.50)	218	0.05 (0.50)
		[0, 15]		[0, 7]
Primary/community service^b^				
General practitioner (visits)	206	3.31 (4.81)	218	3.41 (3.80)
		[0, 48]		[0, 22]
Psychiatrist (visits)	206	2.53 (3.25)	218	1.73 (2.77)
		[0, 19]		[0, 24]
Psychologist (visits)	206	0.80 (3.39)	218	1.42 (8.15)
		[0, 24]		[0, 96]
Dentist (visits)	205	0.55 (1.71)	218	0.70 (1.62)
		[0, 20]		[0,10]
Emergency service (visits)	205	0.08 (0.38)	214	0.09 (0.51)
		[0, 3]		[0, 5]
Other mental health professional (visits)	206	1.77 (5.51)	218	5.41 (17.46)
		[0, 48]		[0, 120]
Other specialist doctor (visits)	205	0.65 (2.56)	218	0.53 (1.44)
		[0, 24]		[0, 12]
Patients’ other costs				
Lost work as physical health (days)	196	1.16 (8.40)	198	0.35 (2.45)
		[0, 90]		[0, 30]
Lost work as mental health (days)	197	2.54 (18.66)	198	1.21 (13.00)
		[0, 180]		[0, 180]
Medicine (euros)	206	237.23 (234.34)	218	243.35 (509.97)
		[0, 1598.10]		[0, 6169.04]

a *N* refers to the number of participants who responded to each question.

b Those contacts do not include care that participants received in the IMPULSE trial.

**Table 3.** Mean costs (euros) for resource use over the first 6 months of the trial by trial group with purchasing power parity adjusted.

	DIALOG+ intervention (*N* = 236)		Standard care (*N* = 232)		Difference (no adjustment)^a^	Difference (with adjustment)^b^
	*N* ^c^	Mean (SD)	*N* ^c^	Mean (SD)	Difference (95% CI)	Difference (95% CI)
Inpatient service						
Voluntary admission to psychiatric hospital (days)	206	52.40	218	32.78	19.62	4.58
		(377.41)		(196.95)	(−30.32,91.52)	(−42.70, 76.63)
Involuntary admission to psychiatric hospital (days)	206	11.89	218	1.30	10.58	11.35
		(96.50)		(13.62)	(0.82, 28.24)	(−0.20, 29.94)
Admission to hospital for physical health (days)	206	7.70	218	1.00	6.69	9.12
		(62.84)		(10.86)	(0.39, 17.30)	(−0.98, 22.68)
**Subtotal**	**206**	**71.98**	**218**	**35.09**	**36.89**	**29.45**
		**(392.17)**		**(197.33)**	**(−13.51, 103.08)**	**(−21.42, 106.93)**
Primary/community service^d^						
General practitioner	206	27.01	218	28.82	−1.81	0.29
		(42.49)		(42.60)	(−9.49, 6.41)	(−6.58, 7.15)
Psychiatrist	206	64.29	218	36.27	28.02*	23.92*
		(128.22)		(68.16)	(10.02, 48.20)	(9.71, 40.64)
Psychologist	206	19.83	218	33.84	−14.01	−19.69
		(80.18)		(185.03)	(−44.21, 7.45)	(−48.72, 5.12)
Dentist	205	8.71	218	15.60	−6.89	−3.75
		(27.02)		(56.77)	(−18.22, −0.03)	(−9.41, 1.56)
Emergency services	205	1.80	214	1.62	0.19	0.31
		(8.48)		(8.92)	(−1.52, 1.83)	(−1.52, 1.84)
Other mental health professional	206	22.02	218	72.11	−50.09*	−52.33*
		(68.62)		(231.14)	(−86.04, −20.00)	(−83.94, −25.13)
Other specialist doctor	205	15.07	218	11.99	3.09	2.70
		(65.81)		(29.94)	(−5.08, 14.01)	(−6.70, 14.87)
**Subtotal**	**205**	**158.63**	**214**	**202.94**	**−44.31**	**−50.08**
		**(202.81)**		**(362.18)**	**(−106.03, 5.60)**	**(−105.06, 3.90)**
Patients’ other costs						
Lost work by patients	196	169.28	198	81.35	87.93	106.81
		(1125.73)		(753.46)	(−107.38, 289.20)	(−84.55, 307.61)
Medication	206	237.23	218	243.35	−6.12	37.03
		(234.34)		(509.97)	(−92.56, 55.65)	(−40.88, 78.90)
DIALOG+/standard care treatments						
DIALOG+ training	236	14.69	–	–	–	–
		(9.13)				
Other staff support for DIALOG+	236	1.24	–	–	–	–
		(2.42)				
Provision of DIALOG+/standard care	236	50.92	232	20.22	–	–
		(62.63)		(21.14)		
Other equipment	236	6.59	232	0.04	–	–
		(9.02)		(0.08)		
Other key resources	236	17.66	232	0.61	–	–
		(10.94)		(1.14)		
**Subtotal**	**236**	**91.11**	**232**	**20.87**	–	–
		**(62.86)**		**(20.71)**		
**Total costs with productivity lost**	**195**	**714.49**	**194**	**584.44**	**130.05**	**154.65**
		**(1247.26)**		**(986.27)**	**(−81.79, 352.24)**	**(−110.94, 422.73)**
**Total costs without productivity lost**	**205**	**565.95**	**214**	**497.78**	**68.17**	**98.42**
		**(516.45)**		**(642.55)**	**(−54.26, 168.60)**	**(−29.49, 208.30)**

a Independent *t*-tests are reported; 95% CI was produced using the bootstrapping method with 1,000 replications; * *p* < 0.05.

b Mixed-effects model with baseline cost and covariates (patients’ age, ICD code, and clinicians’ profession) controlled. 95% CI was produced using bootstrapping replication for 1,000 times with bias corrected. * *p* < 0.05.

c *N* refers to the number of participants who responded to each question.

d Those contacts do not include care that participants received in the IMPULSE trial.

We found differences between the two groups in costs for total resource use over 6 months before randomisation (Supplementary Appendix 2), and these differences were not statistically significant.

### Outcome measures

Table 4 shows the participant level EQ-5D-5L index scores (and estimated QALYs), MANSA scores, and ReQoL-10 sum scores at each assessment time point (baseline and 6 months) by trial group (intervention and control). After adjusting for the baseline differences in EQ-5D-5L index scores and the list of covariates, the mixed-effect model resulted in a difference of 0.0035 QALYs (95% CI –0.0021, 0.0089) between the intervention and control groups over the 6-month period, a difference of 0.1810 points (95% CI 0.0315, 0.3158) for the MANSA, and a difference of 0.7237 points (95% CI –0.2798, 1.9375) for the ReQoL-10. All three outcome measures suggested a health improvement after 6 months of treatment with DIALOG+; however, only the difference in MANSA scores was statistically significant.

**Table 4.** Comparisons of EQ-5D-5L index scores, MANSA scores, and ReQoL-10 sum scores by trial group.

	DIALOG+ intervention (*N* = 236)		Standard care (*N* = 232)		Difference (no adjustment)^a^	Difference (with adjustment)^b^
	*N* ^c^	Mean (SD)	*N* ^c^	Mean (SD)	Difference	Difference
		[min, max]		[min, max]	(95% CI)	(95% CI)
EQ-5D-5L						
Index at baseline	235	0.891 (0.16)	232	0.927 (0.13)	−0.0351*	-
		[0.173, 1]		[0.008, 1]	(−0.0609, −0.0088)	
Index at 6 months	206	0.934 (0.13)	218	0.935 (0.12)	−0.0005	0.0140
		[−0.141, 1]		[0.075, 1]	(−0.0290, 0.0190)	(−0.0083, 0.0355)
QALYs over 6 months^d^	206	0.458 (0.06)	218	0.465 (0.050)	−0.0074	0.0035
		[0.095, 0.5]		[0.195, 0.5]	(−0.0190, 0.0027)	(−0.0021, 0.0089)
MANSA						
At baseline	236	4.480 (0.95)	232	4.537 (0.96)	−0.0576	-
		[1.917, 7]		[1.083, 6.833]	(−0.2304, 0.1242)	
At 6 months	206	4.839 (0.98)	218	4.649 (0.97)	0.1896*	0.1810*
		[2, 6.917]		[1, 7]	(0.0061, 0.3645)	(0.0315, 0.3158)
ReQoL-10						
At baseline	236	25.661 (8.13)	232	25.672 (8.51)	−0.0114	-
		[1, 40]		[2, 40]	(−1.6213, 1.3952)	
At 6 months	206	27.170 (7.88)	218	26.161 (8.31)	1.0094	0.7237
		[2, 40]		[3, 40]	(−0.6621, 2.4348)	(−0.2798, 1.9375)

a Independent *t*-tests are reported; 95% CI was produced using the bootstrapping method with 1,000 replications; * *p* < 0.05.

b Mixed-effects model with baseline outcome measure and covariates (patients’ age, ICD code, and clinicians’ profession) controlled. 95% CI was produced using bootstrapping replication for 1,000 times with bias corrected. * *p* < 0.05.

c N refers to the number of participants who responded to each question.

d Formula used to calculate QALYs over 6 months: QALY = 0.25 X (index at baseline + index at 6 months).

### Cost-effectiveness base case analysis

**Figure 1.**
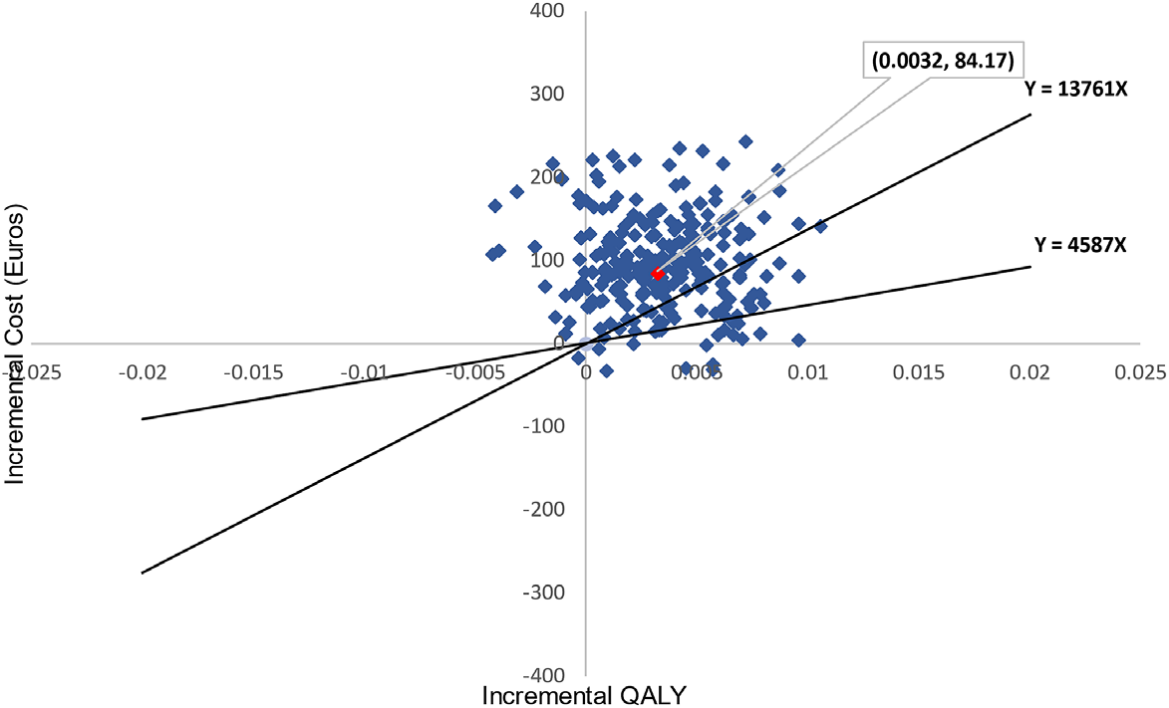
Cost-effectiveness plane (1,000 iterations).

**Figure 2.**
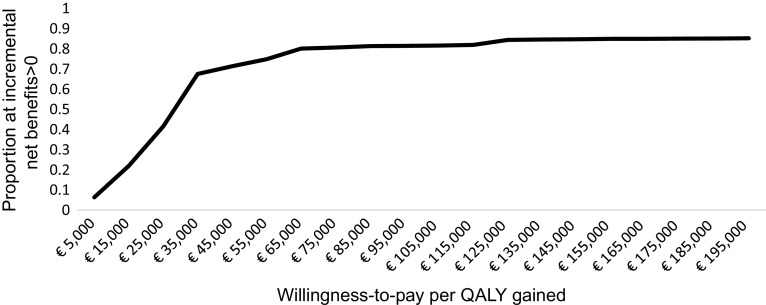
Cost-effectiveness acceptability curve.

Table 5 reports results from the base case evaluation. Cost per QALY gained from implementing DIALOG+ was €26,347.61, achieved by dividing incremental costs of €84.17 (95% CI –8.18, 176.52) by incremental QALYs of 0.0032 (95% CI –0.0015, 0.0079). The weighted GDP per capita was €4,587, and three times this value was €13,761. Figure 1 shows the uncertainty around our point estimate of the ICER using a cost-effectiveness plane, including 1,000 pairs of incremental costs and incremental QALYs from bootstrap replications. Figure 2 presents the cost-effectiveness acceptability curve showing that the probability of DIALOG+ being cost-effective compared with standard care was 3.8% at a willingness-to-pay of €4,587 per QALY, and 18.9% at a willingness-to-pay of €13,761 per QALY. The base case analysis suggested that DIALOG+ was unlikely to be cost-effective.

**Table 5.** Cost-effectiveness analysis for point estimate of the ICER and sensitivity analyses.

	Differences	ICER^a^	One to three times GDP per capita in euros^b^^,^ ^c^
	(95% CI)		
Base case analysis (EQ-5D-5L at 6 months)			
Costs	84.17	€26,347.61	4,587 – 13,761
	(−8.18, 176.52)		
Outcomes	0.0032		
	(−0.0015, 0.0079)		
Sensitivity analysis 1 (complete case analysis)			
Costs	98.42	€28,062.05	4,587 – 13,761
	(−48.08, 244.91)		
Outcomes	0.0035		
	(−0.0031, 0.0101)		
Sensitivity analysis 2 (seemingly unrelated regression)			
Costs	66.09	€19,667.97	4,587 – 13,761
	(−44.86, 177.05)		
Outcomes	0.0034		
	(−0.0024, 0.0091)		
Sensitivity analysis 3.1 (minimum drug price)			
Costs	63.18	€18,649.54	4,587 – 13,761
	(−68.37, 194.73)		
Outcomes	0.0034		
	(−0.0031, 0.0099)		
Sensitivity analysis 3.2 (maximum drug price)			
Costs	78.86	€22,767.93	4,587 – 13,761
	(−71.41, 229.14)		
Outcomes	0.0035		
	(−0.0030, 0.0099)		
Sensitivity analysis 4 (societal perspective)			
Costs	105.48	€31,303.61	4,587 – 13,761
	(−136.19, 347.15)		
Outcomes	0.0034		
	(−0.0031, 0.0099)		
Sensitivity analysis 5 (ReQoL-10 as outcome measure)			
Costs	85.30	€119.02	
	(−45.63, 216.22)		
Outcomes	0.72		
	(−0.4880, 1.9212)		
Sensitivity analysis 6 (MANSA as outcome measure)			
Costs	89.06	€523.53	
	(−41.91, 220.03)		
Outcomes	0.17		
	(0.01, 0.33)		
Sensitivity analysis 7.1^d^			
Bosnia perspective		€22,464.30	4,199 – 12,597
Sensitivity analysis 7.2			
Kosovo (UN Resolution) perspective		Dominant	3,036 – 9,108
Sensitivity analysis 7.3			
Montenegro perspective		€30,514.02	6,124 – 18,372
Sensitivity analysis 7.4			
North Macedonia perspective		€61,293.59	4,139 – 12,417
Sensitivity analysis 7.5			
Serbia perspective		€47,205.13	5,095 – 15,285

a Measure for outcomes was ReQol-10 sum scores in sensitivity analysis 5 and MANSA scores in sensitivity analysis 6. Outcome measures for all other analyses in Table 5 used QALYs.

b For base case analysis and sensitivity analyses 1 to 4, GDP per capita was calculated as the weighted GDP per capita of the five participating countries. The weights were proportions of participants from each country out of the total trial sample size. The formula used was: (€4198.69 *x* 17.31 + €3036.39 *x* 22.01 + €4139.38 x 17.52 + €6123.57 x 26.07 + €5094.54 x 17.09)/100 = €4,587. Three times of the GDP per capita was therefore calculated using €4,587 x 3 = €13,761.

c For sensitivity analyses 7.1 to 7.5, GDP per capita was country-specific.

d For sensitivity analyses 7.1 to 7.5, we ran two regressions for each analysis including a structural cost regression and a QALY outcome regression. Country-perspective ICER was calculated using coefficients from three interactions in terms of the two regressions. We followed the method proposed by Willke et al. (1998).

### Sensitivity analyses

Table 5 reports results from seven sensitivity analyses. The first four sensitivity analyses produced results consistent with the base case analysis: the point estimate of the ICER was above three times the weighted GDP per capita per QALY gained threshold. When ReQoL-10 sum scores were applied as the outcome measure, one score of improvement in ReQoL-10 was associated with additional costs of €119.02 (sensitivity analysis five). Analysis of MANSA scores suggested that an improvement of one score in MANSA was associated with additional costs of €523.53 (sensitivity analysis six). In sensitivity analysis seven, we attempted to estimate country-specific ICERs. DIALOG+ treatment was consistently found not to be cost-effective in four participating countries; Kosovo (UN Resolution) was the only country where the intervention was more effective and less costly than standard care.

## Discussion

The main cost-effectiveness analysis suggested that DIALOG+ is slightly more costly and slightly more effective than standard care over the first 6 months of the trial period. The point estimate of the ICER was higher than the willingness-to-pay value at three times the weighted GDP per capita of the five participating countries. Regarding the uncertainty of this point estimate, our results suggested that the probability was low (18.9%) that DIALOG+ was cost-effective compared with standard care at the provider’s willingness-to-pay threshold. We conducted sensitivity analyses to explore the impact of missing values, estimation methods, key parameters for costs, and evaluation perspectives. None of these analyses challenged the main finding. In country-specific analyses, we found DIALOG+ was more effective and more costly in four of the five participating countries (and the point estimate of the ICER was not cost-effective). Kosovo (UN Resolution) alone showed DIALOG+ as more effective and less costly than standard care. This result should be interpreted with caution as the trial was not powered to detect country-specific treatment effects (in particular, for the EQ-5D-5L measure). Cost analyses shared similar limitations. Additionally, a few unit costs for resource use in Kosovo (UN Resolution) were proxied by the lowest unit price among the other four participating countries due to absence of an official local data source. Country-specific costs for total resource use per participant and outcomes by trial group is reported in Supplementary Appendices 3 and 4, respectively.

In this trial, we observed modest improvements of quality of life measured by three instruments. Only the difference in MANSA scores (i.e., the primary clinical effectiveness outcome in the IMPULSE trial) between the intervention and control groups was statistically significant [14]. The primary economic evaluation relied on QALYs derived from the EQ-5D-5L data as the outcome measure. It should be noted that the EQ-5D-5L has been criticized for its sensitivity regarding people with psychotic disorders and severe and complex nonpsychotic disorders [33]. It has been argued that a condition-specific instrument might be more sensitive in reflecting changes in quality of life in these populations than a generic instrument like the EQ-5D-5L.

DIALOG+ has previously been applied in community care settings in the UK for patients with psychosis [10]. The UK study found that the treatment was less costly than standard care, which was not in line with the results from our IMPULSE study. The study did not collect EQ-5D-5L data, which was one of the limitations reported by its authors. We, therefore, were unable to make a direct comparison between IMPULSE and the UK study of patients’ self-reported EQ-5D-5L and QALYs.

Evidence of cost-effectiveness analyses of treatments for severe mental illness in Southeast Europe is scarce [15]. Treatments are predominantly provided in large psychiatric hospitals with limited community-based alternatives. However, a recently published economic evaluation in the Czech Republic showed that it is cost-effective to discharge patients with chronic psychotic disorders to community care compared with care in psychiatric hospitals [4]. This finding supports one of the aims of introducing DIALOG+ in the LMIC settings, namely, to provide effective and cost-effective mental health treatment for psychotic patients through community-based services.

To our knowledge, this study reports the first cost-effectiveness evaluation of implementing (non-pharmacological) psychosocial treatments for people with psychosis in Southeast Europe. A strength of this study is the trial data that we collected. The challenges around data collection and lack of country-specific unit cost data in multi-country randomised controlled trials are well documented in the literature [29]. It has widely been observed in economic evaluations of multi-country clinical trials that the analyses applied unit costs from one country to all participating countries due to lack of unit cost data from all individual countries [29, 34]. A concern with this approach is around the possibility of generating biased (over/under) estimates for costs. In the IMPULSE trial, we collected resource use and outcomes data at the patient level, as well as country-specific unit costs for each resource item used. This strategy for data collection enabled patient-level data analyses with multi-country costing.

This study has several limitations that should be considered. First, there were no country-specific value sets for the three outcome measures (EQ-5D-5L, MANSA, ReQoL-10). As we observed minimal improvements in QALYs for EQ-5D-5L data, the impact of value set choice on the estimated ICERs could, therefore, be very limited. We reported the results of cost-effectiveness analyses in this paper using ReQoL-10 and MANSA to enable comparisons with future research. Another consideration is around the generalizability of our findings. This issue is well documented for economic evaluations of multi-country randomised controlled trials [29, 35]. We showed different results in cost analyses from the application of the DIALOG+ in the UK [10]. Care should be taken when interpreting our findings to inform decision-making in a different context or/and for a different population. A final limitation of the study relates to the COVID-19 pandemic. The trial was designed to last 12 months, but only the first 6 months of data was interpretable due to disruptions in the study’s delivery relating to pandemic restrictions [14].

Future research might consider producing value sets or conducting mapping exercises to convert scores from MANSA and ReQoL instruments to health utilities in LMIC settings. Furthermore, we found limited research evidence on country-specific cost-effectiveness thresholds in LMICs [36]. The empirical evidence and methodological research in this area are much needed. Finally, we did not find an agreed approach for estimating country-specific cost-effectiveness of an intervention in multi-country clinical trials. Additional research is required in this area in order to inform policy makers regarding resource allocation decisions at the country-specific level.

## Conclusion

This paper reports an economic evaluation of the DIALOG+ intervention alongside the IMPULSE trial. Within the trial, DIALOG+ was shown to be more costly and also more effective for patients with psychosis compared with standard care. The probability of DIALOG+ being a cost-effective treatment at the willingness-to-pay threshold of three times the weighted GDP per capita of the five participating countries was low.

## Trial Registration

ISRCTN11913964.

## Supplementary Materials

To view supplementary material for this article, please visit http://doi.org/10.1192/j.eurpsy.2022.2310.

## Supplementary material

For supplementary material accompanying this paper visit https://doi.org/10.1192/j.eurpsy.2022.2310. click here to view supplementary material

## Abbreviations

CI: confidence interval
CSRI: Client Service Receipt Inventory.
EQ-5D-5L: The 5-level EQ-5D version.
GDP: gross domestic product.
ICD-10: International Classification of Diseases, Tenth Revision.
ICER: incremental cost-effectiveness ratio.
LMICs: low- and middle-income countries.
IMPULSE: Implementation of an effective and cost-effective intervention for patients with psychotic disorders in low and middle-income countries in Southeast Europe.
MANSA: Manchester Short Assessment of Quality of Life.
QALY: Quality-adjusted life year.
ReQoL-10: Recovering Quality of Life, a short 10-item version.
