# Supplementary material for: Cost-effectiveness of implementing a digital psychosocial intervention for patients with psychotic spectrum disorders in low- and middle-income countries in Southeast Europe: Economic evaluation alongside a cluster randomised trial
Source: Eur Psychiatry. 2022 Aug 26;65(1):e56. doi: 10.1192/j.eurpsy.2022.2310 (PMC9532216; doi:10.1192/j.eurpsy.2022.2310)
Supplement: Supplementary file 1 [file S0924933822023100sup001.docx]

**Appendix 1: Summary of main resources and unit costs (euros) by country, adjusted for Purchasing Power Parity**

|  |  | **Bosnia and Herzegovina** | | **Kosovo (UN Resolution)** | | **Montenegro** | | **North Macedonia** | | **Serbia** | |
| --- | --- | --- | --- | --- | --- | --- | --- | --- | --- | --- | --- |
| **Resource item** | **Unit** | **Unit cost**^1^ | **Data source** | **Unit cost**^1^ | **Data source** | **Unit cost**^1^ | **Data source** | **Unit cost**^1^ | **Data source** | **Unit cost**^1^ | **Data source** |
| Psychiatric hospital (voluntary) | Day | 119.82 | HIRI^2,4^ | 21.86 | Proxy^3^ | 21.86 | HIFM^2,5^ | 35.77 | HIFNM^2,6^ | 26.69 | LIS^2,7^ |
| Psychiatric hospital (involuntary) | Day | 119.82 | HIRI | 21.86 | Proxy | 21.86 | HIFM | 35.77 | HIFNM | 26.69 | LIS |
| Physical hospital | Day | 116.69 | HIRI | 21.86 | Proxy | 21.86 | HIFM | 44.71 | HIFNM | 26.69 | LIS |
| General Practitioner | Visit | 10.94 | HIRI | 4.24 | Proxy | 12.04 | HIFM | 4.24 | HIFNM | 4.90 | LIS |
| Psychiatrist | Visit | 16.67 | HIRI | 9.16 | Proxy | 27.85 | HIFM | 74.51 | HIFNM | 9.16 | LIS |
| Psychologist | Visit | 22.40 | HIRI | 21.98 | Proxy | 21.98 | HIFM | 33.53 | HIFNM | 35.40 | LIS |
| Dentist | Visit | 4.69 | HIRI | 4.69 | Proxy | 18.87 | HIFM | 63.33 | HIFNM | 5.57 | LIS |
| Emergency service | Visit | 15.63 | HIRI | 15.63 | Proxy | 17.41 | HIFM | 29.06 | HIFNM | 41.31 | WHO Choice |
| Other mental health professional | Visit | 17.30 | HIRI | 12.46 | Proxy | 12.46 | HIFM | 50.51 | WHO Choice^2,8^ | 43.05 | WHO Choice |
| Other specialist doctor | Visit | 8.17 | HIRI | 8.17 | Proxy | 11.61 | HIFM | 48.49 | WHO Choice | 41.31 | WHO Choice |
| Lost work by patients at baseline | Daily income | 35.81 | World Bank^9^ | 35.81 | Proxy | 57.89 | World Bank | 39.58 | World Bank | 44.22 | World Bank |
| Medicine at baseline | Patient | 407.58 | CRF^2^ | 130.86 | CRF | 286.29 | CRF | 308.31 | CRF | 1393.29 | CRF |
| DIALOG+ clinician | Hourly rate | 12.28 | HEI^2^ | 3.34 | HEI | 16.74 | HEI | 24.44 | HEI | 12.43 | HEI |
| Standard care clinician | Hourly rate | 13.54 | HEI | 3.71 | HEI | 17.37 | HEI | 32.06 | HEI | 9.96 | HEI |

1: Unit costs were Purchasing Power Parity (EU28=1) adjusted.

2: CRF: Case Report Form; HEI: Health Economics Inventory form; HIRI: Health Insurance and Reinsurance Institute of the Federation of Bosnia and Herzegovina; HIFM: Health Insurance Fund of Montenegro; HIFNM: Health Insurance Fund of Republic of North Macedonia; WHO: World Health Organization; LIS: Legal Information System of the Republic of Serbia.

3: In absence of official data source, unit costs for Kosovo (UN Resolution) were derived using the lowest unit price among the other four participating countries in the trial.

4: Data source: <http://www.zzofbih.ba/bs/dokument/tarifnik/68>. Last accessed 12 December 2020.

5: Data source: [https://fzocg.me/davaoci_zdravstvenih_usluga.php?type=prices2.](https://fzocg.me/davaoci_zdravstvenih_usluga.php?type=prices2) Last accessed 12 December 2020.

6: Data source: <http://www.fzo.org.mk/default-en.asp>. Last accessed 12 December 2020.

7: Data source: <http://www.pravno-informacioni-sistem.rs/SlGlasnikPortal/eli/rep/sgrs/drugidrzavniorganiorganizacije/pravilnik/2019/55/2/reg>. Last accessed 12 December 2020.

8: Data source: <https://www.who.int/choice/cost-effectiveness/inputs/health_service/en/>. Last accessed 12 December 2020.

9. Data source: <https://data.worldbank.org/indicator/NY.ADJ.NNTY.PC.CD>. Last accessed 17 February 2021.

## **Appendix 2: Mean costs (euros) for resource use over 6 months before baseline by group, adjusted for Purchasing Power Parity**

|  | **DIALOG+ intervention**  **(N=236)^1^** | | **Standard care**  **(N=232)^1^** | | **Difference**  **(no adjustment)^2^** |
| --- | --- | --- | --- | --- | --- |
|  | N | Mean (SD) | N | Mean (SD) | Difference (P value)  (95% CI) |
| **Inpatient service** | | | | |  |
| Voluntary admission to psychiatric  hospital (days) | 236 | 676.76  (1596.50) | 232 | 392.49  (1511.86) | 284.27  (-5.86, 562.59) |
| Involuntary admission to  psychiatric hospital (days) | 236 | 206.60  (1057.21) | 232 | 96.07  (611.65) | 110.53  (-45.04, 271.58) |
| Admission to hospital for physical  health (days) | 236 | 6.06  (60.17) | 232 | 29.81  (271.44) | -23.75  (-71.56, 3.18) |
| **Sub total** | **236** | **889.42**  **(2387.47)** | **232** | **518.36**  **(1960.70)** | **371.06**  **(-40.63, 762.05)** |
| **Primary/community service^3^** | | | | |  |
| General Practitioner | 234 | 25.52  (40.92) | 231 | 30.77  (38.59) | -5.26  (-12.17, 1.89) |
| Psychiatrist | 232 | 139.45  (231.43) | 231 | 118.09  (138.96) | 21.36  (-11.06, 60.32) |
| Psychologist | 235 | 20.00  (73.99) | 231 | 53.73  (285.27) | -33.73  (-76.16, 1.13) |
| Dentist | 236 | 16.84  (60.90) | 230 | 20.38  (92.52) | -3.54  (-21.63, 9.40) |
| Emergency services | 211 | 2.67  (9.06) | 198 | 2.68  (9.64) | -0.01  (-2.09, 1.74) |
| Other mental health professional | 236 | 42.72  (133.80) | 230 | 52.78  (180.20) | -10.05  (-40.27, 18.87) |
| Other specialist doctor | 236 | 16.86  (49.36) | 232 | 11.30  (29.99) | 5.56  (-1.45, 12.78) |
| **Sub total** | **206** | **211.90**  **(225.61)** | **195** | **264.42**  **(430.25)** | **-52.52**  **(-119.83, 10.15)** |
| **Patients’ other costs** | | | | | |
| Lost work by patients | 232 | 141.19  (813.86) | 230 | 248.61  (1310.80) | -107.42  (-332.78, 76.63) |
| Medicine | 236 | 332.16  (577.65) | 232 | 482.09  (2411.37) | -149.93  (-579.02, 65.93) |
| **Total costs with productivity lost** | **203** | **1640.46 (2789.70)** | **193** | **1633.08**  **(3691.84)** | **7.38**  **(-653.80, 661.01)** |
| **Total costs without productivity lost** | **206** | **1478.06**  **(2628.71)** | **195** | **1357.47**  **(3332.29)** | **120.59**  **(-511.82, 678.01)** |

1: N refers to the number of participants who responded to each question.

2: Independent t-tests are reported; CI was produced using bootstrapping method with 1,000 replications; * P value is <0.05.

3: Those contacts do not include care that participants received in the IMPULSE trial.

## **Appendix 3: Mean costs (euros) for resource use over 6 months before and 6 months after randomisation by country and group, adjusted for Purchasing Power Parity**

|  | **6 months after randomisation** | | **6 months before randomisation** | |
| --- | --- | --- | --- | --- |
|  | **DIALOG+ intervention** | **Standard care** | **DIALOG+ intervention** | **Standard care** |
| Bosnia and Herzegovina | 627.10 | 466.26 | 2327.47 | 2162.24 |
| Kosovo (UN Resolution) | 331.51 | 548.52 | 604.68 | 634.92 |
| Montenegro | 552.87 | 445.20 | 1761.03 | 653.23 |
| North Macedonia | 643.02 | 538.20 | 644.53 | 1462.26 |
| Serbia | 728.87 | 510.20 | 1632.55 | 2444.61 |

## **Appendix 4: Comparisons of EQ-5D-5L index scores, MANSA scores, and ReQoL-10 sum scores by country and group**

|  | **EQ-5D-5L index scores** | | **MANSA scores** | | **ReQoL-10 sum scores** | |
| --- | --- | --- | --- | --- | --- | --- |
|  | **DIALOG+ intervention** | **Standard care** | **DIALOG+ intervention** | **Standard care** | **DIALOG+ intervention** | **Standard care** |
| Bosnia and Herzegovina |  |  |  |  |  |  |
| At baseline | 0.926 | 0.970 | 4.898 | 5.036 | 30.325 | 30.951 |
| At 6 months | 0.964 | 0.961 | 4.901 | 4.912 | 27.757 | 29.769 |
| Kosovo (UN Resolution) |  |  |  |  |  |  |
| At baseline | 0.829 | 0.880 | 4.190 | 4.128 | 23.788 | 23.294 |
| At 6 months | 0.922 | 0.927 | 4.775 | 4.519 | 27.213 | 25.044 |
| Montenegro |  |  |  |  |  |  |
| At baseline | 0.891 | 0.933 | 4.332 | 4.604 | 23.677 | 25.233 |
| At 6 months | 0.932 | 0.942 | 4.650 | 4.654 | 25.378 | 25.632 |
| North Macedonia |  |  |  |  |  |  |
| At baseline | 0.921 | 0.943 | 4.715 | 4.774 | 27.341 | 29.024 |
| At 6 months | 0.950 | 0.948 | 4.979 | 4.732 | 28.600 | 29.171 |
| Serbia |  |  |  |  |  |  |
| At baseline | 0.906 | 0.915 | 4.427 | 4.197 | 24.805 | 20.385 |
| At 6 months | 0.907 | 0.890 | 4.935 | 4.424 | 27.162 | 21.056 |
